# Bis(μ-4-hydr­oxy-2-oxidobenzaldehyde 4-ethyl­thio­semicarbazone)-κ^4^ *O* ^2^,*N* ^1^,*S*:*O* ^2^;κ^4^ *O* ^2^:*O* ^2^,*N* ^1^,*S*-bis­[chloridozinc(II)] dimethyl sulfoxide tris­olvate

**DOI:** 10.1107/S1600536809013385

**Published:** 2009-04-22

**Authors:** Kong Wai Tan, Chew Hee Ng, Mohd Jamil Maah, Seik Weng Ng

**Affiliations:** a Department of Chemistry, University of Malaya, 50603 Kuala Lumpur, Malaysia; b Faculty of Engineering and Science, Universiti Tunku Abdul Rahman, 53300 Kuala Lumpur, Malaysia

## Abstract

The two Zn^II^ atoms in the title compound, [Zn_2_(C_10_H_12_N_3_O_2_S)_2_Cl_2_]·3C_2_H_6_OS, are each *N*,*O*,*S*-chelated by a mono-deprotonated Schiff base ligand. The Zn atoms are bridged through the phenolate O atom, leading to a central Zn_2_O_2_ core. Each Zn atom has a Cl atom in the apical position of a distorted square-pyramidal environment. Hydr­oxy–DMSO (DMSO is dimethyl sulfoxide) O—H⋯O and amide–DMSO N—H⋯O hydrogen bonds link the components of the crystal structure. Two of the DMSO mol­ecules are partially disordered, with each modelled over two sites of equal weight.

## Related literature

For (4-hydr­oxy-2-oxidobenzaldehyde thio­semicarbazonato)­(phenanthroline)zinc DMSO monohydrate, see: Tan *et al.* (2009 ▶).
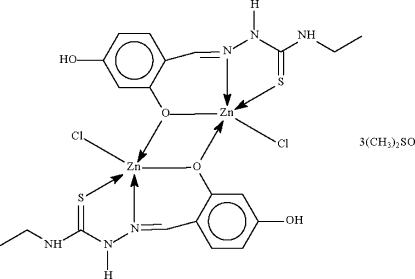


## Experimental

### 

#### Crystal data

[Zn_2_(C_10_H_12_N_3_O_2_S)_2_Cl_2_]·3C_2_H_6_OS
*M* *_r_* = 912.60
Triclinic, 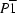

*a* = 9.4151 (1) Å
*b* = 12.4349 (2) Å
*c* = 17.2423 (2) Å
α = 71.4438 (6)°
β = 89.7703 (7)°
γ = 83.4964 (6)°
*V* = 1900.30 (4) Å^3^
*Z* = 2
Mo *K*α radiation
μ = 1.73 mm^−1^
*T* = 123 K
0.25 × 0.20 × 0.20 mm

#### Data collection

Bruker SMART APEX diffractometer
Absorption correction: multi-scan (*SADABS*; Sheldrick, 1996 ▶) *T*_min_ = 0.672, *T*_max_ = 0.724
17632 measured reflections
8627 independent reflections
7501 reflections with *I* > 2σ(*I*)
*R*_int_ = 0.027

#### Refinement

*R*[*F* ^2^ > 2σ(*F* ^2^)] = 0.042
*wR*(*F* ^2^) = 0.134
*S* = 1.00
8627 reflections
450 parameters
16 restraints
H-atom parameters constrained
Δρ_max_ = 1.70 e Å^−3^
Δρ_min_ = −1.03 e Å^−3^

### 

Data collection: *APEX2* (Bruker, 2008 ▶); cell refinement: *SAINT* (Bruker, 2008 ▶); data reduction: *SAINT*; program(s) used to solve structure: *SHELXS97* (Sheldrick, 2008 ▶); program(s) used to refine structure: *SHELXL97* (Sheldrick, 2008 ▶); molecular graphics: *X-SEED* (Barbour, 2001 ▶); software used to prepare material for publication: *publCIF* (Westrip, 2009 ▶).

## Supplementary Material

Crystal structure: contains datablocks I, global. DOI: 10.1107/S1600536809013385/tk2395sup1.cif

Structure factors: contains datablocks I. DOI: 10.1107/S1600536809013385/tk2395Isup2.hkl

Additional supplementary materials:  crystallographic information; 3D view; checkCIF report

## Figures and Tables

**Table 1:** Hydrogen-bond geometry (Å, °)

*D*—H⋯*A*	*D*—H	H⋯*A*	*D*⋯*A*	*D*—H⋯*A*
O2—H2O⋯O5	0.84	1.85	2.623 (3)	153
O4—H4O⋯O6	0.84	1.81	2.645 (4)	171
N2—H2N⋯Cl2^i^	0.88	2.43	3.251 (2)	156
N3—H3N⋯Cl2^i^	0.88	2.51	3.319 (3)	153
N5—H5N⋯O7	0.88	1.90	2.706 (4)	152
N6—H6N⋯O7	0.88	2.05	2.834 (4)	148

Symmetry code: (i) 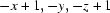.

**Fig. 1.**
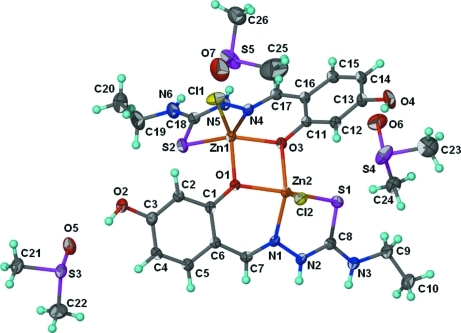
Thermal ellipsoid (Barbour, 2001) plot of Zn2Cl2(C10H12O2N3S)2.3DMSO at the 70% probability level. Hydrogen atoms are drawn as spheres of arbitrary radii. The disorder is not shown.

## supplementary crystallographic information

### Experimental

Zinc chloride (0.14 g, 1 mmol) and 2,4-dihydroxybenzaldehyde 4-ethylthiosemicarbazone (0.24 g, 1 mmol) were heated in ethanol (20 ml) for 3 h. The compound that separated on cooling the solution was recrystallized from a mixture of ethanol and DMSO.

### Refinement

Hydrogen atoms were placed in calculated positions (C–H 0.95 - 0.99 Å, N–H 0.88 Å, O–H 0.84 Å) and were included in the refinement in the riding model approximation, with *U*(H) set to 1.2–1.5*U*(*C*,*N*,*O*).

Two of the three DMSO molecules are disordered. For one of them, only the S5 atom is disordered; the occupancy could not be refined, and was arbitrarily assumed to be 50:50. Pairs of bond lengths involving the unprimed and primed atoms were restrained to within 0.01 Å of each other. The anisotropic displacement factors of the S5 and S5' atoms were restrained to be nearly isotropic. For the other DMSO molecule, only one of the methyl (C23) groups is disordered; the occupancy was also assumed to be 50:50. The two S–C bond lengths involving the unprimed and primed atoms was restrained to within 0.01 Å of each other; their anisotropic displacement factors were similarly restrained.

The final difference Fourier map had peaks/holes in the vicinity of the disordered DMSO.

### Crystal data

**Table:**

[Zn_2_(C_10_H_12_N_3_O_2_S)_2_Cl_2_]·3C_2_H_6_OS	*Z* = 2
*M**_r_* = 912.60	*F*(000) = 940
Triclinic, *P*1	*D*_x_ = 1.595 Mg m^−^^3^
Hall symbol: -P 1	Mo *K*α radiation, λ = 0.71073 Å
*a* = 9.4151 (1) Å	Cell parameters from 9909 reflections
*b* = 12.4349 (2) Å	θ = 2.4–28.3°
*c* = 17.2423 (2) Å	µ = 1.73 mm^−^^1^
α = 71.4438 (6)°	*T* = 123 K
β = 89.7703 (7)°	Block, yellow
γ = 83.4964 (6)°	0.25 × 0.20 × 0.20 mm
*V* = 1900.30 (4) Å^3^	

### Data collection

**Table:**

Bruker SMART APEX diffractometer	8627 independent reflections
Radiation source: fine-focus sealed tube	7501 reflections with *I* > 2σ(*I*)
graphite	*R*_int_ = 0.027
ω scans	θ_max_ = 27.5°, θ_min_ = 1.3°
Absorption correction: multi-scan (*SADABS*; Sheldrick, 1996)	*h* = −12→11
*T*_min_ = 0.672, *T*_max_ = 0.724	*k* = −16→16
17632 measured reflections	*l* = −22→22

### Refinement

**Table:**

Refinement on *F*^2^	Primary atom site location: structure-invariant direct methods
Least-squares matrix: full	Secondary atom site location: difference Fourier map
*R*[*F*^2^ > 2σ(*F*^2^)] = 0.042	Hydrogen site location: inferred from neighbouring sites
*wR*(*F*^2^) = 0.134	H-atom parameters constrained
*S* = 1.00	*w* = 1/[σ^2^(*F*_o_^2^) + (0.0908*P*)^2^ + 2.245*P*] where *P* = (*F*_o_^2^ + 2*F*_c_^2^)/3
8627 reflections	(Δ/σ)_max_ = 0.001
450 parameters	Δρ_max_ = 1.70 e Å^−^^3^
16 restraints	Δρ_min_ = −1.03 e Å^−^^3^

### Fractional atomic coordinates and isotropic or equivalent isotropic displacement parameters (Å^2^)

**Table:**

	*x*	*y*	*z*	*U*_iso_*/*U*_eq_	Occ. (<1)
Zn1	0.58610 (3)	0.43998 (3)	0.218131 (19)	0.01686 (10)	
Zn2	0.56211 (3)	0.18354 (3)	0.330117 (19)	0.01624 (10)	
Cl1	0.79752 (8)	0.47941 (7)	0.15762 (5)	0.03056 (18)	
Cl2	0.76783 (7)	0.05619 (6)	0.36638 (4)	0.02085 (15)	
S1	0.37411 (8)	0.07401 (7)	0.31152 (5)	0.02404 (17)	
S2	0.48125 (9)	0.61073 (6)	0.24461 (5)	0.02341 (17)	
S3	0.92908 (8)	0.61170 (6)	0.65079 (5)	0.02227 (16)	
S4	0.89013 (10)	−0.16673 (8)	0.24447 (6)	0.0391 (2)	
S5	0.0006 (4)	0.7784 (2)	−0.0444 (2)	0.0395 (9)	0.50
S5'	0.0124 (4)	0.7539 (5)	−0.0416 (2)	0.0841 (18)	0.50
O1	0.6071 (2)	0.33696 (17)	0.33727 (12)	0.0182 (4)	
O2	0.8161 (3)	0.5768 (2)	0.45489 (14)	0.0267 (5)	
H2O	0.8365	0.5830	0.5005	0.040*	
O3	0.5635 (2)	0.28127 (17)	0.21268 (12)	0.0207 (4)	
O4	0.6197 (3)	0.0056 (2)	0.07303 (16)	0.0356 (6)	
H4O	0.6906	−0.0198	0.1057	0.053*	
O5	0.8326 (3)	0.6623 (2)	0.57495 (15)	0.0300 (5)	
O6	0.8582 (3)	−0.0784 (2)	0.16274 (17)	0.0405 (6)	
O7	0.1471 (4)	0.7433 (3)	0.0036 (2)	0.0551 (8)	
N1	0.4572 (3)	0.1703 (2)	0.44030 (15)	0.0171 (5)	
N2	0.3612 (3)	0.0906 (2)	0.46242 (15)	0.0189 (5)	
H2N	0.3306	0.0699	0.5127	0.023*	
N3	0.2167 (3)	−0.0284 (2)	0.43583 (16)	0.0213 (5)	
H3N	0.1898	−0.0408	0.4866	0.026*	
N4	0.4198 (3)	0.4913 (2)	0.12552 (15)	0.0186 (5)	
N5	0.3484 (3)	0.5999 (2)	0.11010 (16)	0.0218 (5)	
H5N	0.2887	0.6291	0.0675	0.026*	
N6	0.2969 (3)	0.7645 (2)	0.13923 (17)	0.0278 (6)	
H6N	0.2323	0.7830	0.0990	0.033*	
C1	0.6292 (3)	0.3671 (2)	0.40342 (17)	0.0160 (5)	
C2	0.7112 (3)	0.4557 (2)	0.39834 (17)	0.0182 (5)	
H2	0.7508	0.4935	0.3475	0.022*	
C3	0.7364 (3)	0.4899 (2)	0.46583 (18)	0.0196 (6)	
C4	0.6767 (3)	0.4364 (3)	0.54145 (18)	0.0207 (6)	
H4	0.6929	0.4596	0.5878	0.025*	
C5	0.5945 (3)	0.3498 (3)	0.54691 (18)	0.0203 (6)	
H5	0.5529	0.3145	0.5976	0.024*	
C6	0.5696 (3)	0.3117 (2)	0.48008 (17)	0.0171 (5)	
C7	0.4784 (3)	0.2226 (2)	0.49227 (17)	0.0189 (5)	
H7	0.4302	0.2006	0.5424	0.023*	
C8	0.3138 (3)	0.0440 (2)	0.40855 (18)	0.0182 (5)	
C9	0.1519 (3)	−0.0886 (3)	0.38711 (19)	0.0247 (6)	
H9A	0.2275	−0.1269	0.3617	0.030*	
H9B	0.0891	−0.0334	0.3429	0.030*	
C10	0.0654 (3)	−0.1763 (3)	0.4423 (2)	0.0273 (7)	
H10A	0.1287	−0.2319	0.4849	0.041*	
H10B	0.0200	−0.2157	0.4098	0.041*	
H10C	−0.0084	−0.1380	0.4678	0.041*	
C11	0.5363 (3)	0.2507 (2)	0.14728 (17)	0.0184 (5)	
C12	0.5907 (3)	0.1422 (3)	0.14537 (18)	0.0221 (6)	
H12	0.6471	0.0912	0.1905	0.027*	
C13	0.5629 (4)	0.1082 (3)	0.07780 (19)	0.0241 (6)	
C14	0.4744 (4)	0.1794 (3)	0.01244 (19)	0.0239 (6)	
H14	0.4515	0.1542	−0.0321	0.029*	
C15	0.4212 (3)	0.2864 (3)	0.01366 (18)	0.0224 (6)	
H15	0.3618	0.3352	−0.0310	0.027*	
C16	0.4522 (3)	0.3261 (2)	0.07932 (17)	0.0188 (5)	
C17	0.3931 (3)	0.4403 (3)	0.07340 (17)	0.0200 (6)	
H17	0.3301	0.4812	0.0281	0.024*	
C18	0.3696 (3)	0.6611 (3)	0.15971 (18)	0.0208 (6)	
C19	0.3170 (5)	0.8499 (3)	0.1791 (2)	0.0385 (9)	
H19A	0.4200	0.8585	0.1817	0.046*	
H19B	0.2831	0.8243	0.2357	0.046*	
C20	0.2352 (4)	0.9629 (3)	0.1322 (3)	0.0398 (9)	
H20A	0.2705	0.9890	0.0765	0.060*	
H20B	0.2486	1.0191	0.1597	0.060*	
H20C	0.1333	0.9543	0.1297	0.060*	
C21	1.0107 (4)	0.7267 (3)	0.6644 (2)	0.0279 (7)	
H21A	1.0785	0.7522	0.6209	0.042*	
H21B	1.0617	0.7012	0.7178	0.042*	
H21C	0.9370	0.7901	0.6620	0.042*	
C22	0.8139 (4)	0.5914 (4)	0.7354 (2)	0.0411 (9)	
H22A	0.7611	0.6649	0.7333	0.062*	
H22B	0.8711	0.5599	0.7866	0.062*	
H22C	0.7462	0.5383	0.7326	0.062*	
C23	0.9021 (12)	−0.2911 (7)	0.2100 (7)	0.053 (2)*	0.50
H23A	0.8239	−0.2819	0.1702	0.079*	0.50
H23B	0.8949	−0.3597	0.2570	0.079*	0.50
H23C	0.9940	−0.2989	0.1842	0.079*	0.50
C23'	0.9473 (10)	−0.3045 (6)	0.2467 (6)	0.047 (2)*	0.50
H23D	0.8886	−0.3249	0.2079	0.070*	0.50
H23E	0.9382	−0.3572	0.3020	0.070*	0.50
H23F	1.0476	−0.3096	0.2314	0.070*	0.50
C24	0.7265 (5)	−0.1906 (3)	0.2938 (2)	0.0384 (8)	
H24A	0.6874	−0.1223	0.3070	0.058*	
H24B	0.7426	−0.2556	0.3444	0.058*	
H24C	0.6585	−0.2073	0.2575	0.058*	
C25	−0.0562 (7)	0.6467 (6)	−0.0429 (4)	0.0777 (18)	0.50
H25A	−0.0871	0.6067	0.0120	0.117*	0.50
H25B	0.0233	0.5995	−0.0574	0.117*	0.50
H25C	−0.1361	0.6611	−0.0826	0.117*	0.50
C26	0.0451 (5)	0.8189 (4)	−0.1478 (2)	0.0421 (9)	0.50
H26A	0.0854	0.8917	−0.1625	0.063*	0.50
H26B	−0.0411	0.8273	−0.1820	0.063*	0.50
H26C	0.1157	0.7601	−0.1568	0.063*	0.50
C25'	−0.0562 (7)	0.6467 (6)	−0.0429 (4)	0.0777 (18)	0.50
H25D	−0.0636	0.5963	0.0133	0.117*	0.50
H25E	0.0037	0.6059	−0.0737	0.117*	0.50
H25F	−0.1518	0.6702	−0.0692	0.117*	0.50
C26'	0.0451 (5)	0.8189 (4)	−0.1478 (2)	0.0421 (9)	0.50
H26D	0.1187	0.8703	−0.1533	0.063*	0.50
H26E	−0.0435	0.8624	−0.1763	0.063*	0.50
H26F	0.0778	0.7594	−0.1721	0.063*	0.50

### Atomic displacement parameters (Å^2^)

**Table:**

	*U*^11^	*U*^22^	*U*^33^	*U*^12^	*U*^13^	*U*^23^
Zn1	0.02043 (18)	0.01589 (17)	0.01541 (17)	−0.00603 (12)	0.00188 (12)	−0.00533 (13)
Zn2	0.01946 (18)	0.01542 (17)	0.01495 (17)	−0.00544 (12)	0.00213 (12)	−0.00527 (12)
Cl1	0.0225 (4)	0.0360 (4)	0.0261 (4)	−0.0062 (3)	0.0062 (3)	0.0008 (3)
Cl2	0.0209 (3)	0.0194 (3)	0.0217 (3)	−0.0031 (3)	0.0025 (3)	−0.0055 (3)
S1	0.0277 (4)	0.0297 (4)	0.0190 (3)	−0.0144 (3)	0.0040 (3)	−0.0100 (3)
S2	0.0304 (4)	0.0197 (3)	0.0225 (4)	−0.0024 (3)	−0.0034 (3)	−0.0101 (3)
S3	0.0211 (3)	0.0203 (3)	0.0277 (4)	−0.0065 (3)	0.0029 (3)	−0.0095 (3)
S4	0.0338 (5)	0.0291 (4)	0.0456 (5)	−0.0114 (4)	−0.0118 (4)	0.0033 (4)
S5	0.0408 (16)	0.0392 (11)	0.0362 (15)	0.0319 (12)	−0.0108 (11)	−0.0212 (10)
S5'	0.045 (2)	0.181 (5)	0.0333 (18)	−0.051 (3)	−0.0006 (15)	−0.032 (2)
O1	0.0249 (10)	0.0165 (9)	0.0145 (9)	−0.0064 (8)	0.0014 (8)	−0.0054 (7)
O2	0.0340 (12)	0.0265 (11)	0.0237 (11)	−0.0165 (10)	−0.0001 (9)	−0.0094 (9)
O3	0.0321 (11)	0.0161 (9)	0.0151 (9)	−0.0054 (8)	−0.0010 (8)	−0.0057 (8)
O4	0.0525 (16)	0.0249 (12)	0.0344 (13)	0.0034 (11)	−0.0096 (11)	−0.0191 (11)
O5	0.0316 (12)	0.0311 (12)	0.0325 (12)	−0.0062 (10)	−0.0055 (10)	−0.0163 (10)
O6	0.0447 (15)	0.0339 (14)	0.0351 (14)	−0.0023 (12)	0.0030 (12)	−0.0008 (11)
O7	0.065 (2)	0.0502 (18)	0.0486 (17)	0.0013 (15)	−0.0322 (16)	−0.0156 (14)
N1	0.0181 (11)	0.0153 (11)	0.0187 (11)	−0.0070 (9)	0.0035 (9)	−0.0049 (9)
N2	0.0209 (12)	0.0184 (11)	0.0191 (11)	−0.0091 (9)	0.0053 (9)	−0.0064 (9)
N3	0.0222 (12)	0.0231 (12)	0.0204 (12)	−0.0102 (10)	0.0021 (10)	−0.0071 (10)
N4	0.0210 (12)	0.0171 (11)	0.0191 (11)	−0.0059 (9)	0.0029 (9)	−0.0063 (9)
N5	0.0255 (13)	0.0189 (12)	0.0217 (12)	−0.0021 (10)	−0.0020 (10)	−0.0075 (10)
N6	0.0344 (15)	0.0219 (13)	0.0285 (14)	0.0018 (11)	−0.0041 (11)	−0.0117 (11)
C1	0.0158 (12)	0.0151 (12)	0.0177 (13)	−0.0018 (10)	0.0003 (10)	−0.0063 (10)
C2	0.0192 (13)	0.0178 (13)	0.0187 (13)	−0.0055 (10)	0.0024 (10)	−0.0061 (11)
C3	0.0194 (13)	0.0177 (13)	0.0221 (14)	−0.0044 (10)	−0.0022 (11)	−0.0063 (11)
C4	0.0261 (14)	0.0217 (14)	0.0170 (13)	−0.0050 (11)	−0.0010 (11)	−0.0093 (11)
C5	0.0233 (14)	0.0210 (14)	0.0182 (13)	−0.0040 (11)	0.0014 (11)	−0.0079 (11)
C6	0.0191 (13)	0.0165 (13)	0.0168 (13)	−0.0032 (10)	0.0009 (10)	−0.0063 (10)
C7	0.0207 (13)	0.0200 (14)	0.0162 (13)	−0.0048 (11)	0.0044 (10)	−0.0051 (11)
C8	0.0176 (13)	0.0168 (13)	0.0200 (13)	−0.0034 (10)	0.0014 (10)	−0.0051 (10)
C9	0.0274 (15)	0.0255 (15)	0.0233 (15)	−0.0128 (12)	−0.0020 (12)	−0.0075 (12)
C10	0.0241 (15)	0.0269 (16)	0.0312 (16)	−0.0109 (12)	−0.0042 (13)	−0.0070 (13)
C11	0.0235 (14)	0.0195 (13)	0.0154 (13)	−0.0097 (11)	0.0036 (10)	−0.0075 (11)
C12	0.0305 (15)	0.0182 (14)	0.0192 (14)	−0.0071 (12)	0.0027 (12)	−0.0067 (11)
C13	0.0331 (16)	0.0193 (14)	0.0243 (15)	−0.0076 (12)	0.0047 (12)	−0.0114 (12)
C14	0.0320 (16)	0.0253 (15)	0.0200 (14)	−0.0106 (12)	0.0024 (12)	−0.0125 (12)
C15	0.0263 (15)	0.0252 (15)	0.0183 (13)	−0.0077 (12)	−0.0002 (11)	−0.0088 (12)
C16	0.0224 (14)	0.0183 (13)	0.0184 (13)	−0.0067 (11)	0.0025 (11)	−0.0080 (11)
C17	0.0227 (14)	0.0212 (14)	0.0171 (13)	−0.0065 (11)	0.0007 (11)	−0.0063 (11)
C18	0.0242 (14)	0.0205 (14)	0.0191 (13)	−0.0065 (11)	0.0042 (11)	−0.0072 (11)
C19	0.054 (2)	0.0283 (17)	0.0374 (19)	0.0057 (16)	−0.0094 (17)	−0.0191 (15)
C20	0.042 (2)	0.0292 (18)	0.052 (2)	−0.0032 (15)	0.0022 (18)	−0.0191 (17)
C21	0.0296 (16)	0.0256 (16)	0.0305 (16)	−0.0119 (13)	−0.0042 (13)	−0.0086 (13)
C22	0.038 (2)	0.053 (2)	0.036 (2)	−0.0224 (18)	0.0152 (16)	−0.0144 (18)
C24	0.053 (2)	0.0283 (18)	0.0347 (19)	−0.0110 (16)	0.0034 (17)	−0.0096 (15)
C25	0.061 (3)	0.095 (5)	0.072 (4)	−0.022 (3)	0.019 (3)	−0.015 (3)
C26	0.050 (2)	0.040 (2)	0.0345 (19)	0.0049 (18)	−0.0079 (17)	−0.0133 (16)
C25'	0.061 (3)	0.095 (5)	0.072 (4)	−0.022 (3)	0.019 (3)	−0.015 (3)
C26'	0.050 (2)	0.040 (2)	0.0345 (19)	0.0049 (18)	−0.0079 (17)	−0.0133 (16)

### Geometric parameters (Å, °)

**Table:**

Zn1—O3	2.040 (2)	C4—H4	0.9500
Zn1—O1	2.042 (2)	C5—C6	1.408 (4)
Zn1—N4	2.134 (3)	C5—H5	0.9500
Zn1—Cl1	2.2738 (8)	C6—C7	1.441 (4)
Zn1—S2	2.4144 (8)	C7—H7	0.9500
Zn2—O3	2.004 (2)	C9—C10	1.514 (4)
Zn2—O1	2.041 (2)	C9—H9A	0.9900
Zn2—N1	2.106 (2)	C9—H9B	0.9900
Zn2—Cl2	2.3098 (8)	C10—H10A	0.9800
Zn2—S1	2.4330 (8)	C10—H10B	0.9800
S1—C8	1.704 (3)	C10—H10C	0.9800
S2—C18	1.709 (3)	C11—C12	1.399 (4)
S3—O5	1.512 (2)	C11—C16	1.420 (4)
S3—C21	1.776 (3)	C12—C13	1.395 (4)
S3—C22	1.783 (4)	C12—H12	0.9500
S4—O6	1.492 (3)	C13—C14	1.397 (5)
S4—C23'	1.725 (7)	C14—C15	1.374 (4)
S4—C24	1.766 (4)	C14—H14	0.9500
S4—C23	1.818 (8)	C15—C16	1.415 (4)
S5—O7	1.556 (4)	C15—H15	0.9500
S5—C26	1.755 (5)	C16—C17	1.438 (4)
S5—C25	1.771 (8)	C17—H17	0.9500
S5'—O7	1.463 (5)	C19—C20	1.502 (5)
O1—C1	1.333 (3)	C19—H19A	0.9900
O2—C3	1.349 (3)	C19—H19B	0.9900
O2—H2O	0.8400	C20—H20A	0.9800
O3—C11	1.333 (3)	C20—H20B	0.9800
O4—C13	1.354 (4)	C20—H20C	0.9800
O4—H4O	0.8400	C21—H21A	0.9800
N1—C7	1.291 (4)	C21—H21B	0.9800
N1—N2	1.380 (3)	C21—H21C	0.9800
N2—C8	1.342 (4)	C22—H22A	0.9800
N2—H2N	0.8800	C22—H22B	0.9800
N3—C8	1.335 (4)	C22—H22C	0.9800
N3—C9	1.465 (4)	C23—H23A	0.9800
N3—H3N	0.8800	C23—H23B	0.9800
N4—C17	1.293 (4)	C23—H23C	0.9800
N4—N5	1.384 (3)	C23'—H23D	0.9800
N5—C18	1.341 (4)	C23'—H23E	0.9800
N5—H5N	0.8800	C23'—H23F	0.9800
N6—C18	1.327 (4)	C24—H24A	0.9800
N6—C19	1.466 (4)	C24—H24B	0.9800
N6—H6N	0.8800	C24—H24C	0.9800
C1—C2	1.396 (4)	C25—H25A	0.9800
C1—C6	1.428 (4)	C25—H25B	0.9800
C2—C3	1.390 (4)	C25—H25C	0.9800
C2—H2	0.9500	C26—H26A	0.9800
C3—C4	1.409 (4)	C26—H26B	0.9800
C4—C5	1.374 (4)	C26—H26C	0.9800
			
O3—Zn1—O1	75.75 (8)	N3—C9—H9A	109.9
O3—Zn1—N4	82.80 (9)	C10—C9—H9A	109.9
O1—Zn1—N4	135.57 (9)	N3—C9—H9B	109.9
O3—Zn1—Cl1	103.84 (7)	C10—C9—H9B	109.9
O1—Zn1—Cl1	114.07 (6)	H9A—C9—H9B	108.3
N4—Zn1—Cl1	108.60 (7)	C9—C10—H10A	109.5
O3—Zn1—S2	148.58 (7)	C9—C10—H10B	109.5
O1—Zn1—S2	96.90 (6)	H10A—C10—H10B	109.5
N4—Zn1—S2	81.74 (7)	C9—C10—H10C	109.5
Cl1—Zn1—S2	106.97 (3)	H10A—C10—H10C	109.5
O3—Zn2—O1	76.57 (8)	H10B—C10—H10C	109.5
O3—Zn2—N1	142.48 (9)	O3—C11—C12	119.6 (3)
O1—Zn2—N1	84.26 (8)	O3—C11—C16	121.4 (3)
O3—Zn2—Cl2	110.95 (7)	C12—C11—C16	119.0 (3)
O1—Zn2—Cl2	108.05 (6)	C13—C12—C11	120.6 (3)
N1—Zn2—Cl2	105.53 (7)	C13—C12—H12	119.7
O3—Zn2—S1	95.96 (6)	C11—C12—H12	119.7
O1—Zn2—S1	145.34 (6)	O4—C13—C12	121.6 (3)
N1—Zn2—S1	81.81 (7)	O4—C13—C14	117.6 (3)
Cl2—Zn2—S1	106.22 (3)	C12—C13—C14	120.9 (3)
C8—S1—Zn2	96.09 (10)	C15—C14—C13	118.8 (3)
C18—S2—Zn1	96.70 (10)	C15—C14—H14	120.6
O5—S3—C21	106.24 (15)	C13—C14—H14	120.6
O5—S3—C22	105.80 (18)	C14—C15—C16	122.0 (3)
C21—S3—C22	97.28 (18)	C14—C15—H15	119.0
O6—S4—C23'	117.6 (4)	C16—C15—H15	119.0
O6—S4—C24	107.80 (18)	C15—C16—C11	118.6 (3)
C23'—S4—C24	98.9 (3)	C15—C16—C17	117.3 (3)
O6—S4—C23	97.2 (4)	C11—C16—C17	124.2 (3)
C23'—S4—C23	23.5 (4)	N4—C17—C16	124.5 (3)
C24—S4—C23	95.6 (4)	N4—C17—H17	117.8
O7—S5—C26	104.6 (3)	C16—C17—H17	117.8
O7—S5—C25	104.0 (3)	N6—C18—N5	115.7 (3)
C26—S5—C25	94.9 (3)	N6—C18—S2	121.2 (2)
C1—O1—Zn1	127.72 (17)	N5—C18—S2	123.1 (2)
C1—O1—Zn2	129.08 (17)	N6—C19—C20	110.2 (3)
Zn1—O1—Zn2	102.82 (9)	N6—C19—H19A	109.6
C3—O2—H2O	109.5	C20—C19—H19A	109.6
C11—O3—Zn2	126.62 (18)	N6—C19—H19B	109.6
C11—O3—Zn1	128.58 (18)	C20—C19—H19B	109.6
Zn2—O3—Zn1	104.24 (9)	H19A—C19—H19B	108.1
C13—O4—H4O	109.5	C19—C20—H20A	109.5
S5'—O7—S5	10.9 (3)	C19—C20—H20B	109.5
C7—N1—N2	115.9 (2)	H20A—C20—H20B	109.5
C7—N1—Zn2	127.8 (2)	C19—C20—H20C	109.5
N2—N1—Zn2	116.14 (17)	H20A—C20—H20C	109.5
C8—N2—N1	121.1 (2)	H20B—C20—H20C	109.5
C8—N2—H2N	119.4	S3—C21—H21A	109.5
N1—N2—H2N	119.4	S3—C21—H21B	109.5
C8—N3—C9	124.5 (3)	H21A—C21—H21B	109.5
C8—N3—H3N	117.8	S3—C21—H21C	109.5
C9—N3—H3N	117.8	H21A—C21—H21C	109.5
C17—N4—N5	115.4 (3)	H21B—C21—H21C	109.5
C17—N4—Zn1	127.1 (2)	S3—C22—H22A	109.5
N5—N4—Zn1	116.46 (18)	S3—C22—H22B	109.5
C18—N5—N4	120.4 (3)	H22A—C22—H22B	109.5
C18—N5—H5N	119.8	S3—C22—H22C	109.5
N4—N5—H5N	119.8	H22A—C22—H22C	109.5
C18—N6—C19	124.4 (3)	H22B—C22—H22C	109.5
C18—N6—H6N	117.8	S4—C23—H23A	109.5
C19—N6—H6N	117.8	S4—C23—H23B	109.5
O1—C1—C2	119.8 (2)	S4—C23—H23C	109.5
O1—C1—C6	121.7 (2)	S4—C23'—H23D	109.5
C2—C1—C6	118.5 (3)	S4—C23'—H23E	109.5
C3—C2—C1	121.6 (3)	H23D—C23'—H23E	109.5
C3—C2—H2	119.2	S4—C23'—H23F	109.5
C1—C2—H2	119.2	H23D—C23'—H23F	109.5
O2—C3—C2	117.4 (3)	H23E—C23'—H23F	109.5
O2—C3—C4	122.5 (3)	S4—C24—H24A	109.5
C2—C3—C4	120.2 (3)	S4—C24—H24B	109.5
C5—C4—C3	118.7 (3)	H24A—C24—H24B	109.5
C5—C4—H4	120.6	S4—C24—H24C	109.5
C3—C4—H4	120.6	H24A—C24—H24C	109.5
C4—C5—C6	122.4 (3)	H24B—C24—H24C	109.5
C4—C5—H5	118.8	S5—C25—H25A	109.5
C6—C5—H5	118.8	S5—C25—H25B	109.5
C5—C6—C1	118.6 (3)	H25A—C25—H25B	109.5
C5—C6—C7	117.6 (3)	S5—C25—H25C	109.5
C1—C6—C7	123.8 (2)	H25A—C25—H25C	109.5
N1—C7—C6	125.0 (3)	H25B—C25—H25C	109.5
N1—C7—H7	117.5	S5—C26—H26A	109.5
C6—C7—H7	117.5	S5—C26—H26B	109.5
N3—C8—N2	115.8 (3)	H26A—C26—H26B	109.5
N3—C8—S1	121.7 (2)	S5—C26—H26C	109.5
N2—C8—S1	122.5 (2)	H26A—C26—H26C	109.5
N3—C9—C10	109.1 (3)	H26B—C26—H26C	109.5
			
O3—Zn2—S1—C8	−152.58 (12)	S2—Zn1—N4—N5	11.49 (18)
O1—Zn2—S1—C8	−77.64 (15)	C17—N4—N5—C18	−179.2 (3)
N1—Zn2—S1—C8	−10.33 (12)	Zn1—N4—N5—C18	−9.7 (3)
Cl2—Zn2—S1—C8	93.54 (10)	Zn1—O1—C1—C2	−33.5 (4)
O3—Zn1—S2—C18	−70.75 (16)	Zn2—O1—C1—C2	154.7 (2)
O1—Zn1—S2—C18	−144.63 (12)	Zn1—O1—C1—C6	145.8 (2)
N4—Zn1—S2—C18	−9.46 (12)	Zn2—O1—C1—C6	−26.0 (4)
Cl1—Zn1—S2—C18	97.56 (11)	O1—C1—C2—C3	179.8 (3)
O3—Zn1—O1—C1	−179.4 (2)	C6—C1—C2—C3	0.5 (4)
N4—Zn1—O1—C1	−115.8 (2)	C1—C2—C3—O2	−179.5 (3)
Cl1—Zn1—O1—C1	81.5 (2)	C1—C2—C3—C4	−1.1 (4)
S2—Zn1—O1—C1	−30.5 (2)	O2—C3—C4—C5	178.8 (3)
O3—Zn1—O1—Zn2	−5.97 (8)	C2—C3—C4—C5	0.4 (4)
N4—Zn1—O1—Zn2	57.69 (15)	C3—C4—C5—C6	1.0 (5)
Cl1—Zn1—O1—Zn2	−104.99 (8)	C4—C5—C6—C1	−1.6 (4)
S2—Zn1—O1—Zn2	142.92 (7)	C4—C5—C6—C7	−178.3 (3)
O3—Zn2—O1—C1	179.4 (2)	O1—C1—C6—C5	−178.4 (3)
N1—Zn2—O1—C1	31.9 (2)	C2—C1—C6—C5	0.9 (4)
Cl2—Zn2—O1—C1	−72.6 (2)	O1—C1—C6—C7	−2.0 (4)
S1—Zn2—O1—C1	98.5 (2)	C2—C1—C6—C7	177.3 (3)
O3—Zn2—O1—Zn1	6.05 (9)	N2—N1—C7—C6	−177.5 (3)
N1—Zn2—O1—Zn1	−141.47 (10)	Zn2—N1—C7—C6	7.3 (4)
Cl2—Zn2—O1—Zn1	114.05 (7)	C5—C6—C7—N1	−172.4 (3)
S1—Zn2—O1—Zn1	−74.86 (13)	C1—C6—C7—N1	11.1 (5)
O1—Zn2—O3—C11	−178.1 (3)	C9—N3—C8—N2	−179.8 (3)
N1—Zn2—O3—C11	−116.8 (2)	C9—N3—C8—S1	−0.2 (4)
Cl2—Zn2—O3—C11	77.4 (2)	N1—N2—C8—N3	−177.2 (2)
S1—Zn2—O3—C11	−32.5 (2)	N1—N2—C8—S1	3.3 (4)
O1—Zn2—O3—Zn1	−6.10 (9)	Zn2—S1—C8—N3	−172.0 (2)
N1—Zn2—O3—Zn1	55.23 (18)	Zn2—S1—C8—N2	7.5 (3)
Cl2—Zn2—O3—Zn1	−110.56 (8)	C8—N3—C9—C10	171.2 (3)
S1—Zn2—O3—Zn1	139.53 (8)	Zn2—O3—C11—C12	−41.2 (4)
O1—Zn1—O3—C11	178.0 (3)	Zn1—O3—C11—C12	148.7 (2)
N4—Zn1—O3—C11	37.2 (2)	Zn2—O3—C11—C16	138.4 (2)
Cl1—Zn1—O3—C11	−70.3 (2)	Zn1—O3—C11—C16	−31.7 (4)
S2—Zn1—O3—C11	98.2 (2)	O3—C11—C12—C13	179.6 (3)
O1—Zn1—O3—Zn2	6.12 (9)	C16—C11—C12—C13	0.0 (4)
N4—Zn1—O3—Zn2	−134.67 (11)	C11—C12—C13—O4	177.0 (3)
Cl1—Zn1—O3—Zn2	117.88 (8)	C11—C12—C13—C14	−3.2 (5)
S2—Zn1—O3—Zn2	−73.63 (15)	O4—C13—C14—C15	−176.7 (3)
C26—S5—O7—S5'	−86.0 (14)	C12—C13—C14—C15	3.5 (5)
C25—S5—O7—S5'	13.0 (13)	C13—C14—C15—C16	−0.6 (5)
O3—Zn2—N1—C7	−81.3 (3)	C14—C15—C16—C11	−2.6 (4)
O1—Zn2—N1—C7	−22.2 (3)	C14—C15—C16—C17	178.8 (3)
Cl2—Zn2—N1—C7	85.0 (3)	O3—C11—C16—C15	−176.8 (3)
S1—Zn2—N1—C7	−170.4 (3)	C12—C11—C16—C15	2.8 (4)
O3—Zn2—N1—N2	103.6 (2)	O3—C11—C16—C17	1.7 (4)
O1—Zn2—N1—N2	162.6 (2)	C12—C11—C16—C17	−178.6 (3)
Cl2—Zn2—N1—N2	−90.20 (19)	N5—N4—C17—C16	−178.0 (3)
S1—Zn2—N1—N2	14.45 (18)	Zn1—N4—C17—C16	13.8 (4)
C7—N1—N2—C8	169.5 (3)	C15—C16—C17—N4	−175.0 (3)
Zn2—N1—N2—C8	−14.7 (3)	C11—C16—C17—N4	6.5 (5)
O3—Zn1—N4—C17	−27.9 (2)	C19—N6—C18—N5	−172.4 (3)
O1—Zn1—N4—C17	−89.0 (3)	C19—N6—C18—S2	8.1 (5)
Cl1—Zn1—N4—C17	74.4 (3)	N4—N5—C18—N6	179.5 (3)
S2—Zn1—N4—C17	179.6 (3)	N4—N5—C18—S2	−1.0 (4)
O3—Zn1—N4—N5	164.0 (2)	Zn1—S2—C18—N6	−171.6 (3)
O1—Zn1—N4—N5	102.9 (2)	Zn1—S2—C18—N5	9.0 (3)
Cl1—Zn1—N4—N5	−93.72 (19)	C18—N6—C19—C20	170.8 (3)

### Hydrogen-bond geometry (Å, °)

**Table:**

*D*—H···*A*	*D*—H	H···*A*	*D*···*A*	*D*—H···*A*
O2—H2O···O5	0.84	1.85	2.623 (3)	153
O4—H4O···O6	0.84	1.81	2.645 (4)	171
N2—H2N···Cl2^i^	0.88	2.43	3.251 (2)	156
N3—H3N···Cl2^i^	0.88	2.51	3.319 (3)	153
N5—H5N···O7	0.88	1.90	2.706 (4)	152
N6—H6N···O7	0.88	2.05	2.834 (4)	148

Symmetry codes: (i) −*x*+1, −*y*, −*z*+1.
